# Age-related decline in circulating IGF-1 associates with impaired neurovascular coupling responses in older adults

**DOI:** 10.1007/s11357-022-00623-2

**Published:** 2022-07-23

**Authors:** Luca Toth, Andras Czigler, Emoke Hegedus, Hedvig Komaromy, Krisztina Amrein, Endre Czeiter, Andriy Yabluchanskiy, Akos Koller, Gergely Orsi, Gabor Perlaki, Attila Schwarcz, Andras Buki, Zoltan Ungvari, Peter J. Toth

**Affiliations:** 1 Department of Neurosurgery, Medical School, University of Pecs, 2 Ret Street, Pecs, 7624 Hungary; 2 Institute for Translational Medicine, Medical School, University of Pecs, Pecs, Hungary; 3 Vascular Cognitive Impairment and Neurodegeneration Program, Oklahoma Center for Geroscience and Healthy Brain Aging, Department of Biochemistry and Molecular Biology, University of Oklahoma Health Sciences Center, Oklahoma City, OK USA; 4 ELKH-PTE Clinical Neuroscience MR Research Group, Eötvös Lóránd Research Network (ELKH), Pecs, Hungary; 5 Department of Neurology, Medical School, University of Pecs, Pecs, Hungary; 6 Department of Health Promotion Sciences, College of Public Health, University of Oklahoma Health Sciences Center, Oklahoma City, OK USA; 7 The Peggy and Charles Stephenson Cancer Center, University of Oklahoma Health Sciences Center, Oklahoma City, OK 73104 USA; 8 International Training Program in Geroscience, Doctoral School of Basic and Translational Medicine/Department of Public Health, Semmelweis University, Budapest, Hungary

**Keywords:** Neurovascular uncoupling, Cognitive decline, Vascular cognitive impairment, VCI, VCID, Aging

## Abstract

Impairment of moment-to-moment adjustment of cerebral blood flow (CBF) to the increased oxygen and energy requirements of active brain regions via neurovascular coupling (NVC) contributes to the genesis of age-related cognitive impairment. Aging is associated with marked deficiency in the vasoprotective hormone insulin-like growth factor-1 (IGF-1). Preclinical studies on animal models of aging suggest that circulating IGF-1 deficiency is causally linked to impairment of NVC responses. The present study was designed to test the hypotheses that decreases in circulating IGF-1 levels in older adults also predict the magnitude of age-related decline of NVC responses. In a single-center cross-sectional study, we enrolled healthy young (*n* = 31, 11 female, 20 male, mean age: 28.4 + / − 4.2 years) and aged volunteers (*n* = 32, 18 female, 14 male, mean age: 67.9 + / − 4.1 years). Serum IGF-1 level, basal CBF (phase contrast magnetic resonance imaging (MRI)), and NVC responses during the trail making task (with transcranial Doppler sonography) were assessed. We found that circulating IGF-1 levels were significantly decreased with age and associated with decreased basal CBF. Age-related decline in IGF-1 levels predicted the magnitude of age-related decline in NVC responses. In conclusion, our study provides additional evidence in support of the concept that age-related circulating IGF-1 deficiency contributes to neurovascular aging, impairing CBF and functional hyperemia in older adults.

## Introduction

Age-related impairment of neurovascular coupling responses (NVC; or “functional hyperemia”) importantly contributes to the pathogenesis of vascular cognitive impairment and dementia (VCID) in older adults [1]. Impaired NVC responses compromise the rapid adjustment of cerebral blood flow to the increased oxygen and energy requirements of active brain regions and hinder the washout of toxic metabolic by-products [1], thereby promoting neuronal dysfunction. The mechanisms by which aging impairs neurovascular coupling responses are multifaceted and likely involve a significant reduction in endothelial production of nitric oxide (NO) [2–4] and astrocyte dysfunction [1]. Preclinical studies demonstrate that increased cellular oxidative stress and mitochondrial dysfunction contribute to neurovascular dysfunction associated with old age [1, 5–8].

There is growing evidence, including findings obtained in experiments using heterochronic parabiosis in mice as a model system, that age-related changes in circulating anti-geronic factors importantly contribute to the regulation of vascular aging processes and the genesis of endothelial dysfunction and oxidative stress in a non–cell autonomous manner [9]. Insulin-like growth factor-1 (IGF-1) is an anabolic peptide hormone produced by the liver, which exerts multifaceted vasoprotective and anti-geronic effects [1, 10–36]. Circulating IGF-1 significantly decreases with age in humans and in laboratory animals due to an age-related decline in GH production/release [17, 35, 37–40]. Importantly, preclinical studies demonstrate that circulating IGF-1 deficiency in transgenic mouse models impairs both endothelium-mediated and astrocyte-dependent NVC responses, mimicking the aging phenotype [14, 41]. Additionally, disruption of IGF1R signaling specifically in endothelial cells (*VE-Cadherin-Cre*^*ERT2*^*/Igf1r*^*f/f*^) or astrocytes (*GFAP-Cre*^*ERT2*^*/Igf1r*^*f/f*^) significantly impairs NVC responses in mice, similar to the effects of circulating IGF-1 deficiency and aging [42, 43]. These findings provide strong evidence that circulating IGF-1 modulates NVC responses in the murine brain. Despite these advances, the role of age-related IGF-1 deficiency in neurovascular dysfunction in older adults remains to be determined.

The present study was designed to test the hypotheses that decreases in circulating IGF-1 levels in older adults predict the magnitude of age-related decline of NVC responses. To test this hypothesis in a single-center cross-sectional study, we enrolled healthy young (18–40 years old) and aged (≥ 60 years old) subjects. Serum IGF-1 level, basal CBF (phase contrast MRI), and NVC responses during the trail making task (transcranial Doppler sonography) were assessed. The relationship between circulating IGF-1 and the functional parameters was analyzed.

## Methods

### Patient enrolment

This study was approved by the National Ethics Committee (6552–4/2020/EUIG) in Hungary. The patients were prospectively enrolled into the study on a voluntary basis. The inclusion criteria were 18–40 and over 60 years of age and a written informed consent from each study participant. The exclusion criteria were previous or ongoing neurological disease; any condition that could affect IGF-1 levels, including neoplasia, nephrectomy, renal disease, endocrine disorder, pregnancy, uncontrolled diabetes mellitus, starvation, and corticosteroid therapy; and non-penetrable acoustic window in the temporal area. Total of 63 participants were enrolled in this study, with 31 young (mean age: 28.4 ± 4.2 years, 11 female, 20 male) and 32 older adults (mean age: 67.9 ± 4.1 years, 18 female, 14 male).

### Measurement of IGF-1 levels

Blood samples were collected before assessment of NVC responses, centrifuged at 3500 revolutions per minute for 15 min and processed for serum collection. IGF-1 levels were determined by enzyme-amplified chemiluminescence immunoassay (CLIA) using an IGF-1 assay kit (Siemens Healthcare Diagnostics, Los Angeles, USA, catalogue nr: L2KGF2) on a Siemens IMMULITE 2000 platform (Siemens Healthcare GmbH, Erlangen, Germany).

### Measurement of NVC in humans

To evaluate NVC responses, a transcranial Doppler ultrasound system (DWL Multi-Dop® T digital, Singen, Germany) with 2-MHz transducers was used. The transducers were fixed bilaterally to the temporal acoustic windows and middle cerebral arteries’ (MCA) flow velocities were registered at the depth of 45–60 mm. Continuous beat-to-beat blood pressure and end-tidal carbon dioxide levels were recorded. The raw data of flow measurements were collected and analyzed by ICM + ® software (Cambridge, England). NVC responses were assessed during the performance of 60-s long trail making task, with respective 60-s resting state. Trail making test is a widely accessible neuropsychological test, in which the participant has to connect 25 encircled numbers in numerical order by pen. The numbers are semi-randomly placed in the test to prevent crossing lines. For the evaluation, both the time needed to perform the test and the mistakes are evaluated [44]. Breath-hold tests were performed as the positive control examinations, during which the participants were asked to hold their breath for 45 s, or as long as they can, as previously described [45]. To assess the cerebral blood flow and NVC responses, the following values were continuously registered: arterial blood pressure (ABP), mean arterial pressure (MAP), and mean cerebral blood flow velocity (CBFv) on both sides measured in the middle of MCAs as cm/s. Cerebrovascular conductance index (CVCi) was calculated for the right and left MCA as the quotient of CBFv and MAP in cm/s/mmHg to eliminate the effect of blood pressure change on the cerebral blood flow variation [46].

During neurovascular tests, baseline mean arterial blood pressure assessed by a finger-cuff noninvasive continuous blood pressure monitor (CNAP Monitor 500 HD, CNSystems, Graz, Austria) was not significantly different between the age groups (90 ± 15 vs. 107 ± 16 mmHg in young and aged persons, respectively). CO_2_ levels, assessed with a capnograph (Promed, Kwun Tong, Kowloon, Hong Kong), were in the physiologic range (35–45 mmHg) during the measurements.

### Magnetic resonance imaging (MRI): flow analysis

MRI measurements were performed on a 3 T MRI scanner (MAGNETOM Prisma^Fit^, Siemens Healthcare, Erlangen, Germany) with a 20-channel head/neck coil. The flow was measured in the M1 segments of both left and right middle cerebral arteries (MCAs) using a parasagittal two-dimensional single-slice phase contrast (PC) sequence with peripheral pulse gating and the following parameters: TR/TE = 89.22/9.03 ms, flip angle = 15 degrees, slice thickness = 4 mm, FOV = 140 × 140 mm^2^, matrix size = 256 × 256 interpolated to 512 × 512, receiver bandwidth = 130 Hz/pixel, averages = 3, number of phases = 25, and velocity encoding (VENC) = 100 cm/s in through-plane direction. The imaging plane was arranged perpendicular to the longitudinal axis of the vessels (Fig. 2) using a native 3D time-of-flight MR angiography (TOF-MRA) with the following parameters: TR/TE = 22/3.86 ms, flip angle = 18 degrees, slice thickness = 0.7 mm, FOV = 167 × 222 mm^2^, matrix size = 202 × 384, receiver bandwidth = 178 Hz/pixel, 4 overlapping (27.08%) slabs, and a total of 153 axial slices (48 slices/slab). Flow analysis of PC MRI data were performed using Argus software (Leonardo workstation; Siemens Healthcare, Erlangen, Germany). After loading the magnitude, phase, and rephased images into Argus, vessel contour of MCA was manually outlined on the first cardiac phase image and then automatically propagated to all other cardiac phases. The propagated contours (i.e., automatically outlined contours) were carefully checked for each cardiac phase and manually adjusted to ensure accurate vessel boundary delineation (Fig. 2A, B). To avoid phase aliasing near vessel borders, velocity range was adjusted from ± 100 cm/s to − 50/ + 150 cm/s. After applying background correction, average flow (ml/s) and average MCA area (cm^2^) were automatically calculated by the software.

### Statistical analysis

Statistical analysis was carried out by unpaired t test. The bivariate Pearson correlation was used to measure the strength and direction of linear relationships between pairs of continuous variables. GraphPad Pro and SPSS were used to perform the statistical tests. A *p* value less than 0.05 was considered statistically significant.

## Results

### Circulating IGF-1 level is significantly decreased in aging and correlates with age

**Fig. 1:**
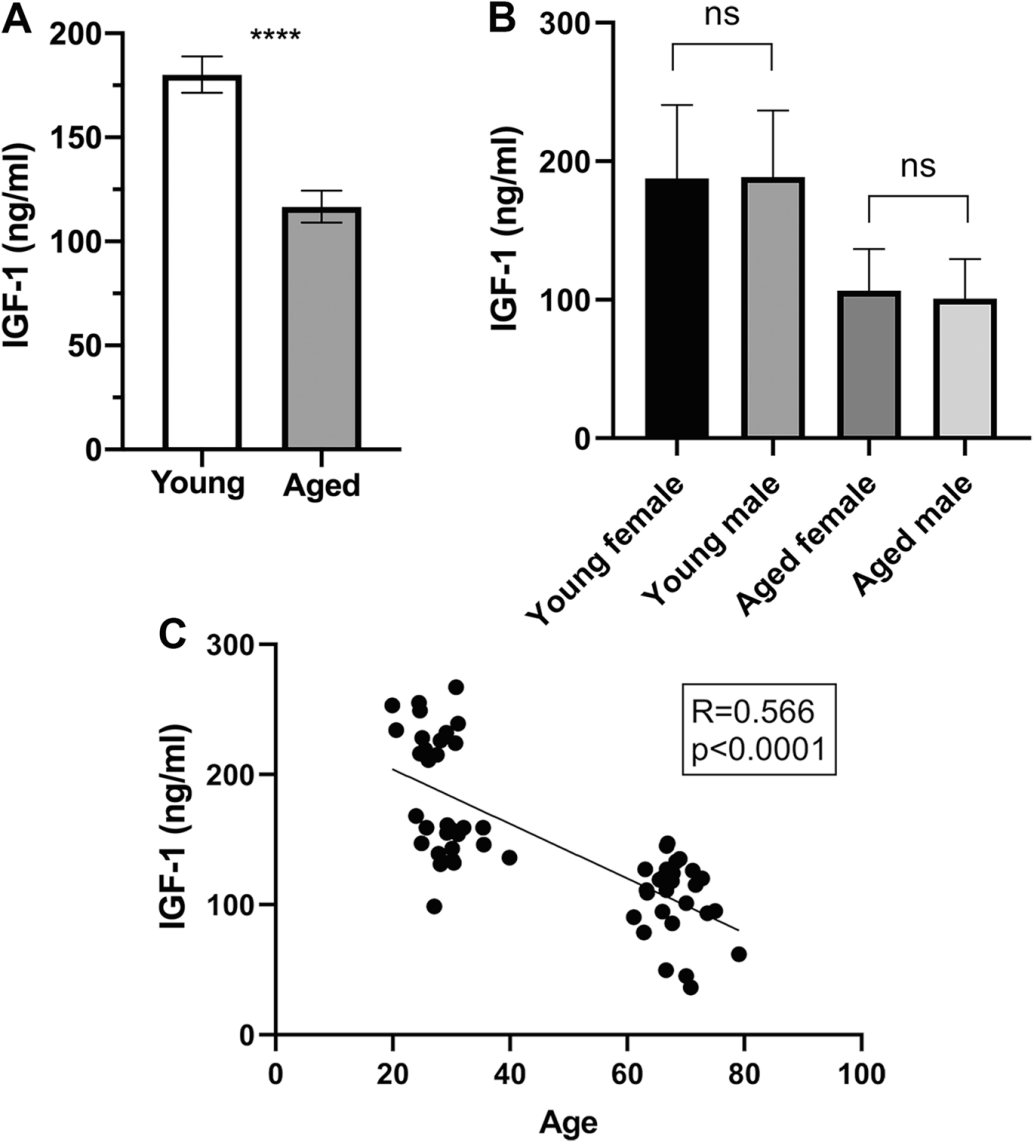
Circulating IGF-1 level is significantly decreased in aged study participants. **A** Serum IGF-1 levels (ng/ml) are significantly decreased in aged study participants (*n* = 32), as compared to young persons (*n* = 31). Data are mean ± S.E.M. ****p < 0.0001. **B** We did not observe sex-related differences in circulating IGF-1 levels (ng/ml) in the studied groups (*n* = 11 and 20 in young females and males, respectively, and *n* = 11 and 20 in older females and males, n.s.: nonsignificant). **C** Correlation between serum IGF-1 levels (ng/ml) and age (years) in young (*n* = 31) and aged (*n* = 32) study participants

We found that serum IGF-1 levels (ng/ml) are significantly decreased in aged study participants, as compared to young persons (Fig. 1). Importantly, we did not observe sex-related differences in circulating IGF-1 levels in the studied groups, and serum IGF-1 levels significantly correlated with age (Fig. 1). In basic cardio-cerebrovascular risk factors of study participants, we found that mean arterial blood pressure was significantly higher in older participants than in young volunteers; however, older participants were not hypertensive (Table 1.) We also found that smoking was significantly more frequent among young persons (Table 1). Blood glucose metabolism and cholesterol levels were not different between the study groups (Table 1).

**Table 1:** Characteristics of study participants. Basic cardio-cerebrovascular risk factors of study participants are summarized (MAP, mean arterial blood pressure). Significant differences were found in MAP (^*^p < 0.0001 vs. young) and smoking (^#^*p* = 0.0337 vs. aged). Participants with diabetes mellitus were on oral medication, and their blood sugar levels and hemoglobin a1c levels were in the normal range

		Young group	Aged group
*n*		31	32
Age (in years)		28.37 + / − 4.2	67.9 + / − 4.1
Sex	Female	11	18
	Male	20	14
Baseline MAP (mean + / − SD in Hgmm)		90.66 + / − 14.8	107.05 + / − 16.24^*^
Baseline heart rate (beat/min)		69.02 + / − 8.9	67.25 + / − 10.56
Smoking	Yes	25%^#^	6%
	No	75%	94%
Diabetes mellitus	Yes	3%	15%
	No	97%	85%
Total cholesterol level (mean + / − SD in mmol/l)		4.73 + / − 0.83	5.69 + / − 3.3
Low density lipoprotein level (mean + / − SD in mmol/l)		2.61 + / − 0.72	3.49 + / − 2.88

### NVC responses are impaired in aged individuals

**Fig. 2:**
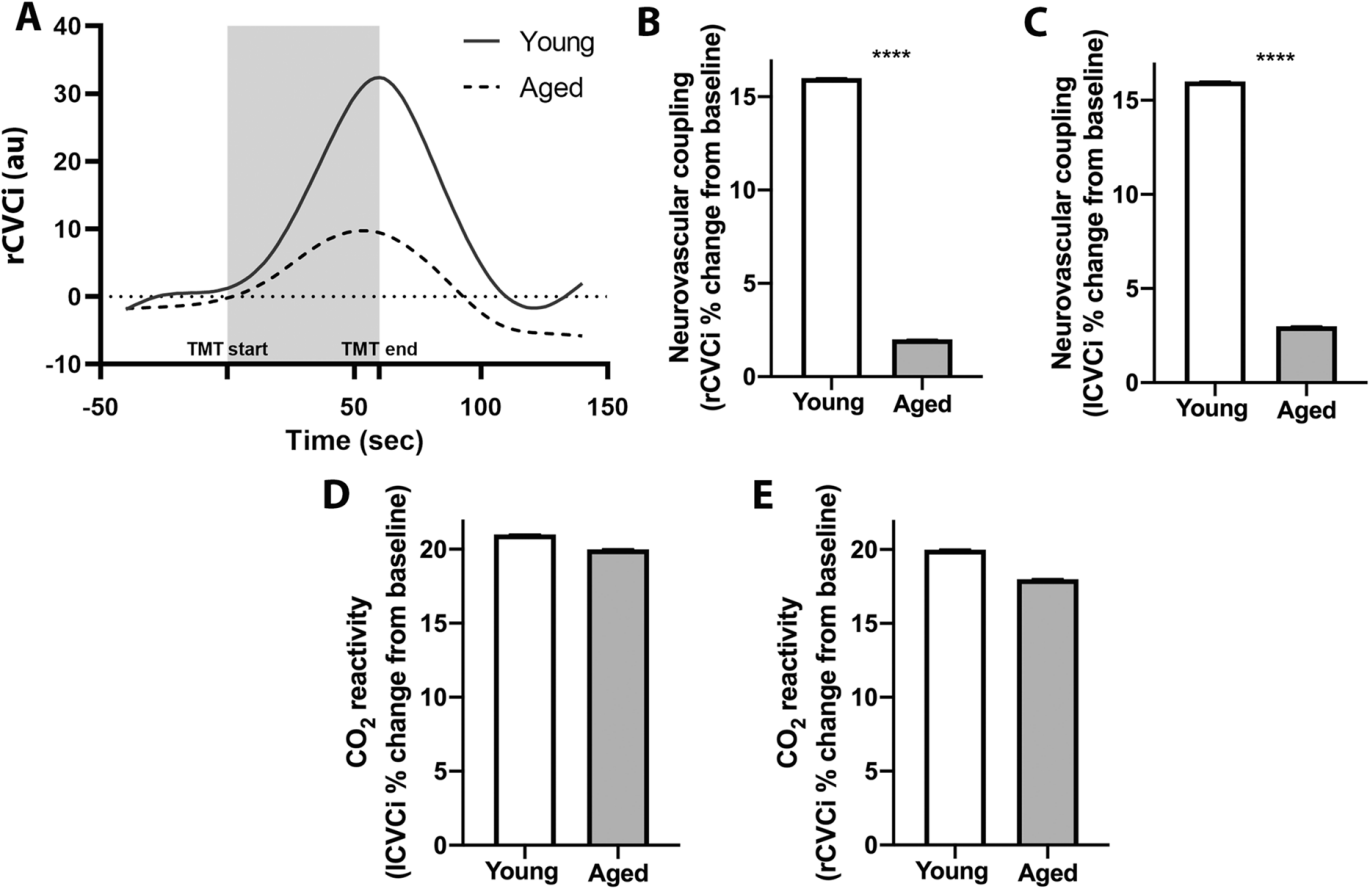
Impaired neurovascular coupling (NVC) responses in aging. **A** Representative trace of changes of the right cerebral vascular conductance index (CVCi) during trail making test (TMT) in a 25-year-old (young) and a 65-year-old (aged) study participant. The gray area represents the period while the TMT test was performed. The task-evoked maximal NVC response in the young and old study participant was 32.4% and 9.7%, respectively, as compared to baseline. **B–C** Bar graphs are summary data of NVC responses in young (*n* = 31) and aged (*n* = 32) persons, expressed as percentage changes CVCi in the right (rCVCi, **B**) and left (lCVCi, **C**) middle cerebral arteries. Data are mean ± S.E.M. (young, *n* = 31; aged *n* = 32), ****p < 0.0001.***p < 0.003. **D–E** CO_2_ reactivity in the studied young and aged groups, expressed as percentage changes in CVCi in the left (**D**) and right (**E**) middle cerebral arteries during breath-hold test. Data are mean + / − S.E.M

NVC responses were assessed by calculating the changes in the cerebrovascular conductance index (CVCi) in young and older subjects in response to performing the trail making test. CVCi significantly increased over the baseline levels both in the right and left MCA during the trail making test in both groups (Fig. 2A). We found that NVC responses were significantly decreased in older subjects as compared to young participants (Fig. 1B–C). The breath-hold test also induced significant increases in CVCi in both age groups; however, the difference between the two groups was not statistically significant (Fig. 2D, E).

### Aging decreases basal cerebral blood flow

**Fig. 3:**
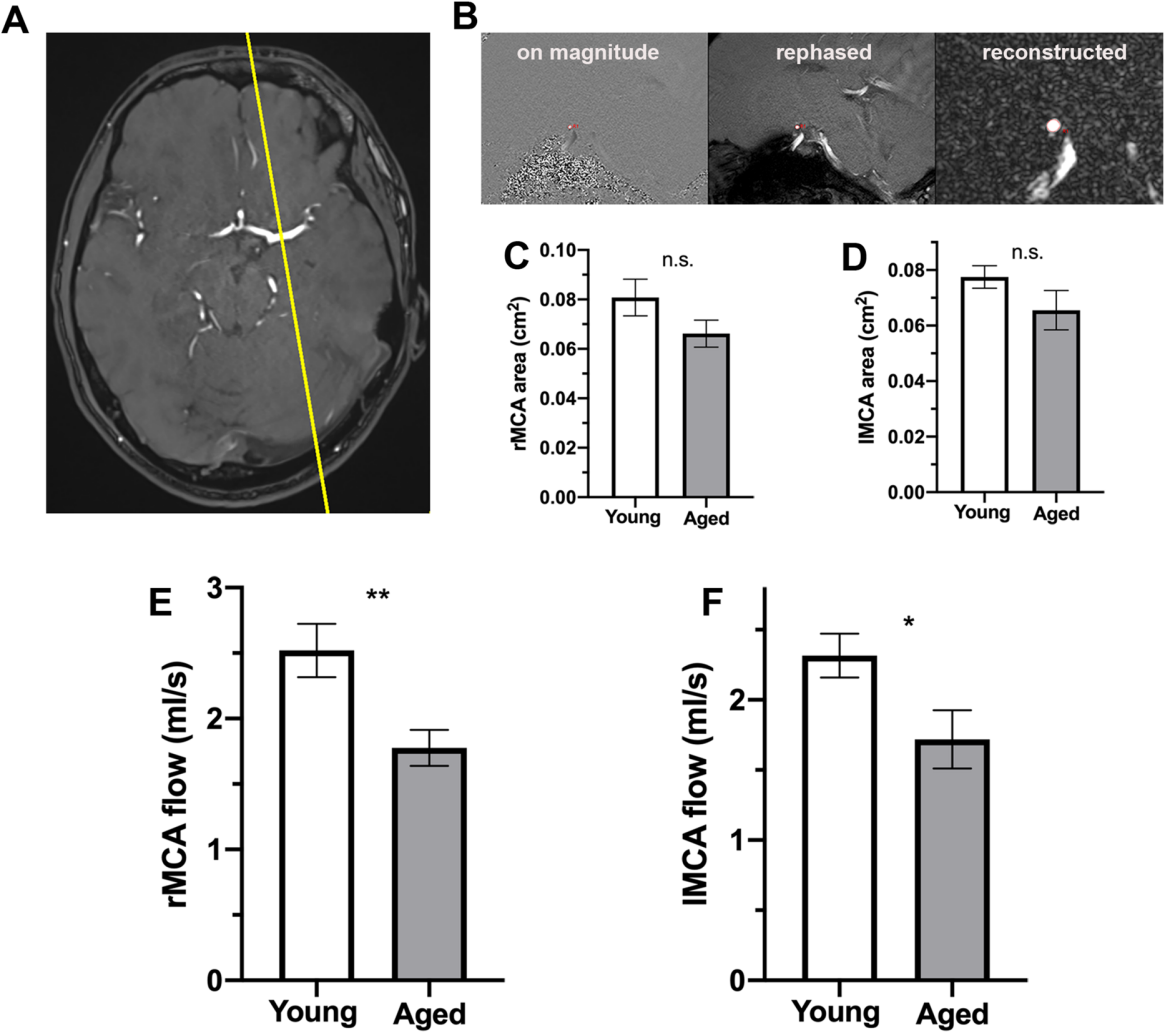
Aging decreases basal cerebral blood flow, measured in the middle cerebral arteries by phase contrast magnetic resonance imaging (MRI) in humans. **A** Representative MRI image of the brain of a 65-year-old study participant. The yellow line shows parasagittal cross section of the left middle cerebral artery (MCA), where the baseline flow velocity was measured on time-of-flight sequence. **B** Representative figure showing magnitude, rephased, and reconstructed sections of the left MCA in parasagittal view. The red circle with hyperintense signal shows the cross section of MCA, and the small red circle shows the reference point in the cerebral parenchyma. **C–D** The bar graphs show that the mean area (cm.^2^) of the right (**C**) and left (**D**) MCA is nonsignificantly decreased in aged (*n* = 12) as compared to young (*n* = 9) people. Data are mean ± S.E.M. **E–F** Mean flow velocity (ml/s) in the right (**E**) and left (**F**) MCAs of young (*n* = 12) and aged (*n* = 9) study participants. Data are mean ± S.E.M.*p < 0.05, **p < 0.003

The mean cross-sectional areas of the right and left MCAs were not significantly different between the two groups (Fig. 3). We found that mean flow velocities in both MCAs were significantly decreased in older subjects as compared to those in younger subjects (Fig. 3E–F).

### Decreased serum IGF-1 levels predict decreased basal blood flow in middle cerebral arteries

**Fig. 4:**
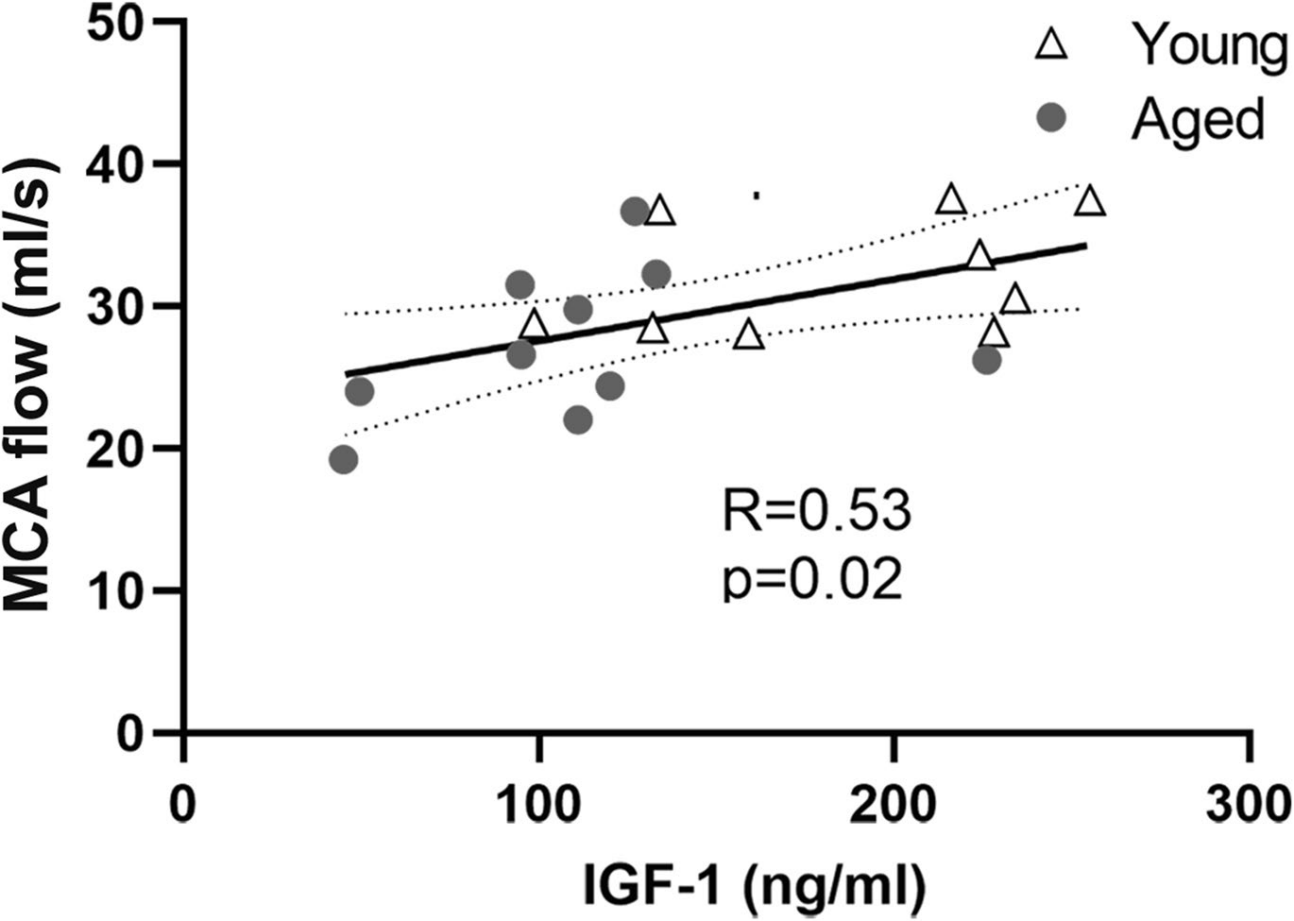
Circulating IGF-1 predicts blood flow velocity in middle cerebral arteries of young and aged adults. Correlation between serum IGF-1 levels and blood flow velocity (ml/s) in middle cerebral arteries (MCA) measured by phase contrast magnetic resonance imaging in young (*n* = 10) and aged (*n* = 11) study participants

We found that serum IGF-1 levels were significantly decreased in older subjects as compared to young participants (Fig. 4A). Serum IGF-1 levels significantly correlated with basal blood flow velocity in middle cerebral arteries in the young and aged study participants (Fig. 4B).

### Decreased serum IGF-1 levels predict impaired NVC responses

**Fig. 5:**
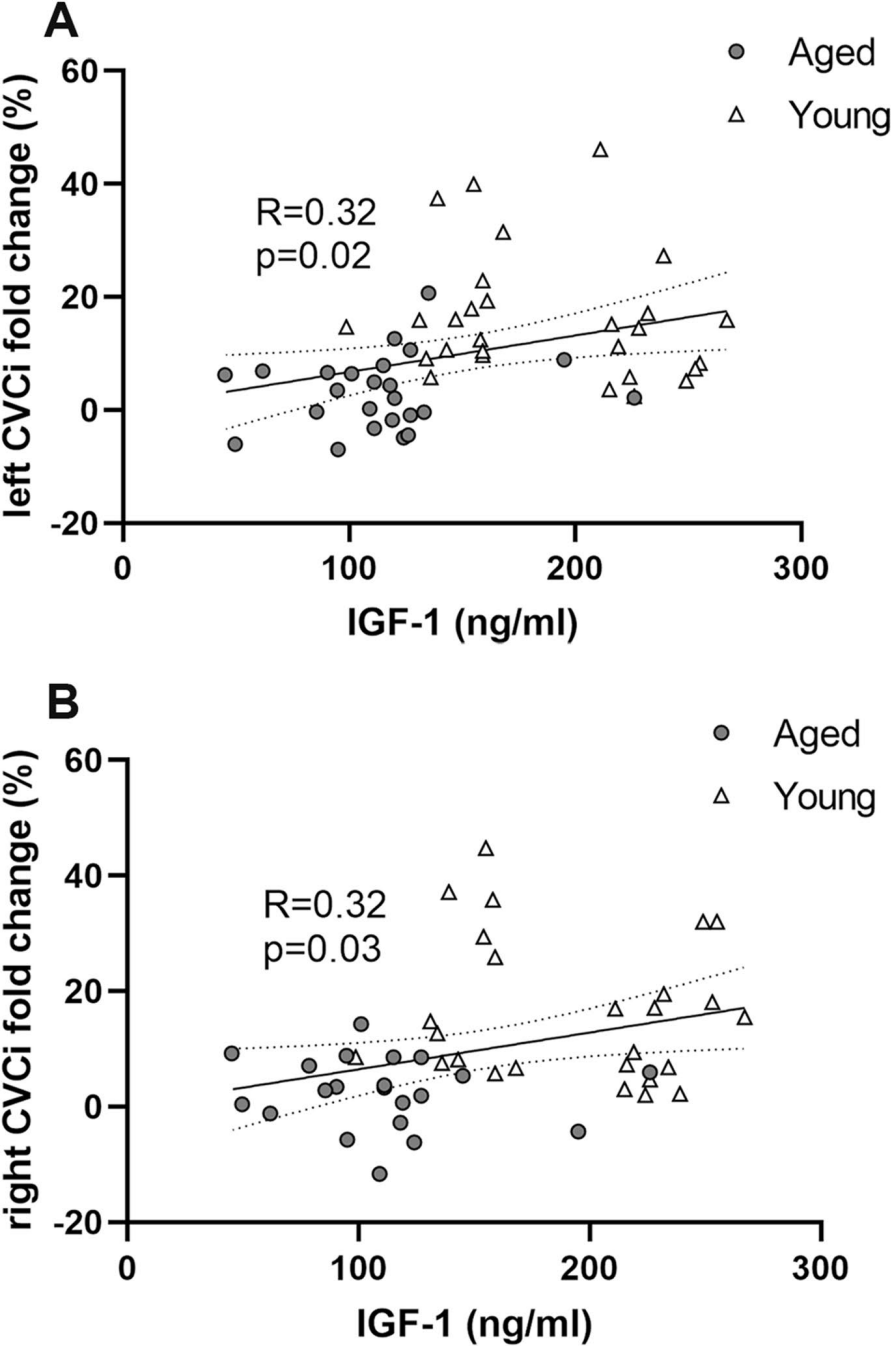
Circulating IGF-1 predicts the magnitude of neurovascular coupling (NVC) responses. **A–B** Correlation between serum IGF-1 levels and NVC responses (expressed as % changes in cerebral vascular conductance index [CVCi] during trail making test measured by transcranial doppler sonography in the left (**B**) and right (**C**) middle cerebral arteries (MCA)) in young (*n* = 31) and aged (*n* = 32) study participants

Serum IGF-1 levels significantly correlated with changes in CVCi in both MCAs during trail making test in the study participants (Fig. 5A–B), indicating the serum IGF-1 levels are associated with NVC responses in an age-dependent manner.

## Discussion

The major findings of our study are that advanced age is associated with impaired NVC responses in humans and that age-related decreases of circulating IGF-1 levels predict the magnitude of age-related decline in functional hyperemia.

Here we show that in healthy older adults, NVC associated with performing the trail making test is attenuated compared to young controls. Functional magnetic resonance imaging (fMRI) studies showed that the trail making test, which provides information on visual search speed, scanning, speed of processing, mental flexibility, as well as executive functioning, evokes NVC responses predominantly in the frontal lobe as well as the middle and superior temporal gyrus [47]. These brain regions are supplied by the MCAs; thus, the TCD-based method used in our study proved to be useful to detect age-related changes in NVC responses evoked by the trail making test. Our study extends the findings of previous investigations demonstrating age-related impairment of NVC responses in older adults evoked and assessed by other methodologies including fMRI, fNIRS, and dynamic retinal vessel analysis [48–51]. Age-related impairment of NVC responses is a universal phenomenon, which is also manifested in laboratory animals [1, 49, 52]. We also confirmed that age-related impairment of NVC responses is also associated with decreased basal cerebral blood flow, suggesting shared etiology (e.g., endothelial dysfunction [53, 54]). Experimental studies suggest that aging is associated with cerebromicrovascular rarefaction [55–57], which likely also attenuates cerebral blood flow. Age-related damage of the neurovascular unit, especially injury of capillary endothelial cells and pericytes, both of which have been suggested to contribute to basal cerebral blood flow as well as neurovascular coupling can underly our clinical observations. From this point, it is significant that recent studies demonstrated that brain capillaries can serve as an active sensory web that converts changes in external K^+^ into rapid, “inside-out” electrical signaling to direct blood flow to active brain regions contributing to functional hyperemia [58]. Dysfunction of this mechanisms due to age-related endothelial dysfunction may contribute to the impaired NVC responses we observed. Future studies should establish the role of alterations of cell adhesion molecule and related capillary stalling [59, 60], alteration of tight junction coverage and associated hypoperfusion [61], and changes of pericytes in age-related dysregulation of cerebral blood flow in humans in a larger cohort of participants.

NVC is essential for maintaining healthy brain function. There is strong evidence that age-related impairment of NVC responses contributes to the genesis of cognitive decline associated with old age [1]. Preclinical studies using pharmacological or genetic approaches to disrupt NVC provide further direct evidence that a causal link exists between neurovascular uncoupling and impaired cognition and gait function in mice [3, 5, 52, 62, 63]. Interestingly, we found that certain older participants in our cohort exhibited inversion of NVC, which may further exacerbate age-related decreases in cerebral blood flow and related cognitive deficits. Inversion of NVC has been previously observed both in animal models and in older patients with diverse age-related pathological conditions, including Parkinson’s disease [64–68]. The diagnostic potential of demonstration of inverse neurovascular coupling responses by TCD in older adults needs to be explored in future studies. This is the first study to provide translational evidence that age-related decline in circulating IGF-1 levels may contribute to the disruption of NVC responses in older adults, extending abundant preclinical findings [14, 41–43]. The mechanisms by which IGF-1 deficiency may disrupt NVC responses in aging are likely multifaceted. Endothelial release of nitric oxide has a key role both in NVC responses and the upstream conduction and spreading of microvascular dilation [3, 63]. IGF-1 receptors are abundantly expressed on endothelial cells [69]. Importantly, both cell-specific disruption of IGF1R signaling in endothelial cells and genetically induced circulating IGF-1 deficiency impair the endothelium-dependent component of NVC responses [14, 43]. The cellular mechanisms by which disruption of endothelial IGF-1/IGF1R signaling impairs endothelium-mediated NVC responses likely include decreased NO bioavailability due to increased production of reactive oxygen species [14]. Mediation of NVC responses also includes astrocyte activation induced by neurotransmitters released from firing neurons and consequential astrocytic release of vasodilator mediators [70–73]. Gliotransmitters that contribute to NVC responses include vasodilator metabolites of arachidonic acid (e.g., epoxyeicosatrienoic acids [EETs] produced by cytochrome P450 enzymes [74–76] and prostaglandins produced by cyclooxygenases). Previous studies showed that both experimentally induced circulating IGF-1 deficiency [14] and cell-specific disruption of IGF1R signaling in astrocytes [42] impair astrocyte-mediated NVC responses. The cellular mechanisms by which disruption of astrocytic IGF-1/IGF1R signaling impairs NVC responses likely include dysregulation of metabotropic glutamate receptors, NMDA receptors, and glutamate transporters [14, 17], upregulation of soluble epoxy hydrolase (which eliminates vasodilator EETs produced during astrocyte activation), and altered expression of cytochrome P450 enzymes [14]. As a result, circulating IGF-1 deficiency leads to an impaired production and increased elimination of astrocyte-derived vasodilators mediating NVC and/or an increased production of vasoconstrictor arachidonic acid metabolites (e.g., 20-hydroxytrienoic acid [20-HETE]) in preclinical models [1, 42]. On the basis of the aforementioned experimental evidence, we posit that increased production of vasoconstrictor eicosanoids, including 20-HETE, may contribute to the inversion of NVC responses observed in some of the older study participants in the present study. In order to modulate astrocyte-derived factors of NVC, IGF-1 has to cross the blood–brain barrier (BBB). It has been suggested that IGF-1 enters the brain by a saturable transport system at the BBB synchronized with IGF-binding proteins to regulate the availability of IGF-1 [77]. Age-related changes of this transport system and its role in local IGF-1 deficiency should be studied in the future. Additionally, IGF-1 deficiency may also promote pathological remodeling of the microvascular network [13], contributing to age-related decreases in basal cerebral blood flow.

The present study has important limitations that need to be acknowledged. First, our present data are correlative in nature. Although our previous preclinical studies suggest that a causal link exists between circulating IGF-1 deficiency and impairment of NVC responses, ascertaining the cause-and-effect relationship between age-related IGF-1 deficiency and impaired functional hyperemia in humans requires additional evidence. For example, it would be interesting to test how treatments that increase circulating IGF-1 levels (e.g., GH replacement) impact NVC responses in older adults. Second, the differential effects of IGF-1 deficiency on astrocyte- and endothelium-dependent NVC responses in older adults should be determined. Third, although TCD is a reliable method to study NVC responses during cognitive tasks [78–81], other methods (fNIRS, fMRI) have better spatiotemporal resolution. The importance of potential regional differences in NVC responses and their regulation by IGF-1 needs further investigation. Also, in experimental murine models, multiple age-related mechanisms appear to act synergistically to impair NVC responses, including increased cellular senescence in the neurovascular unit [6], mitochondrial oxidative stress [8], NAD + deficiency, and impaired SIRT1 signaling [5, 82]. These pathways are especially important players in cardio-cerebrovascular dysfunction due to risk factors such as hypertension, diabetes, and obesity, responsible for the so-called cerebrovascular aging phenotype. Further studies are warranted to understand the interaction between these pathways and age-related IGF-1 deficiency.

### Perspectives

In conclusion, our findings demonstrate that age-related decline in circulating IGF-1 associates with impaired neurovascular coupling responses in older adults. These results add to the growing evidence that IGF-1 exerts important neurovascular protective effects, which likely supports multiple aspects of brain health. In particular, there is strong experimental and translation evidence linking impaired NVC responses to impaired performance on cognitive tasks [1, 5, 52, 62]. Thus, further studies are warranted to determine how the neurovascular alterations caused by decreased circulating IGF-1 levels impact different domains of cognition in older adults. IGF-1 levels and IGF-1 receptors are affected in various pathological conditions. Among the most important ones are traumatic brain injury and diabetes mellitus. Also, age-related insulin resistance may profoundly affect the effects IGF-1 in aged individuals. Further studies should establish the IGF-1–related changes of NVC responses in these conditions. Because impaired NVC responses have also been linked to changes in gait coordination both in preclinical models [52] and older adults [80], future studies should also determine how IGF-1–related changes in NVC relate to subclinical alterations in gait. Determination of gait variability and gait entropy should be quite informative in that regard. Future studies should also investigate how changes in circulating IGF-1 levels in response to various antiaging interventions relate to NVC responses, microvascular endothelial function, and cognitive- and gait-related outcome measures.
